# Surgical Stabilization of Rib Fractures: Current Evidence and Considerations for Posterior Rib Fractures

**DOI:** 10.5761/atcs.ra.26-00069

**Published:** 2026-06-20

**Authors:** I Komang Renteg Sumaarsana, I Gusti Agung Made Adnyana Putra, Ni Made Aprilia Sari

**Affiliations:** 1 Department of Thoracic, Cardiac and Vascular Surgery, Klungkung General Hospital, Klungkung, Bali, Indonesia; 2 Department of Physical Medicine and Rehabilitation, Klungkung General Hospital, Klungkung, Bali, Indonesia

**Keywords:** posterior rib fractures, surgical stabilization of rib fractures, flail chest, thoracic trauma, postoperative rehabilitation

## Abstract

**Purpose:**

Posterior rib fractures represent severe thoracic trauma and are associated with a higher risk of complications due to their proximity to vital intrathoracic structures, including the intercostal vessels and lungs. Surgical stabilization of rib fractures (SSRF) has emerged as an operative strategy to restore chest wall stability, improve respiratory mechanics, and reduce pain. However, evidence specifically addressing posterior rib fractures remains limited, and current understanding is largely extrapolated from studies involving general rib fracture populations.

**Methods:**

This literature review evaluates the principles, operative techniques, effectiveness, and limitations of SSRF in posterior rib fractures, with an emphasis on surgical timing, technical challenges, and postoperative rehabilitation.

**Results:**

Current evidence suggests that SSRF may reduce respiratory complications and improve outcomes in selected patients. Studies report reductions in pneumonia incidence and up to a 95% decrease in tracheostomy requirements in patients with flail chest. Surgical timing also influences outcomes, with pneumonia reported in 18% of patients undergoing SSRF within 72 h compared with 58% in those receiving delayed stabilization.

**Conclusions:**

SSRF may provide clinical benefits in appropriately selected patients with rib fractures. However, evidence specifically addressing posterior rib fractures remains limited, and further high-quality studies are required to clarify optimal indications and surgical strategies.

## Introduction

Rib fractures are a common clinical condition that may result from either traumatic or nontraumatic causes. Most rib fractures occur due to direct penetrating trauma or blunt trauma to the chest. Rib fractures may also be pathological, particularly as a consequence of metastatic cancer originating from other organs. In certain cases, rib fractures can lead to vascular injury, pulmonary parenchymal laceration, and hemopneumothorax, all of which may require specific medical or surgical interventions. In patients with multiple rib fractures, flail chest (FC) may develop, a life-threatening condition that disrupts normal respiratory mechanics and therefore requires prompt intervention.^1–3)^

Rib fractures may occur in the anterior, lateral, or posterior regions of the thoracic cage. Posterior rib fractures are associated with a higher risk of complications, including pneumothorax, hemothorax, atelectasis, and injury to intrathoracic organs. Although surgical intervention such as surgical stabilization of rib fractures (SSRF) is often considered the primary treatment option, certain cases of rib fractures, including posterior rib fractures, may initially be managed with nonoperative management (NOM), particularly in the absence of chest wall instability or significant respiratory complications.^4)^

The SSRF approach involves the use of specialized plates and screws, intramedullary devices, or other fixation techniques to restore the contour and stability of the thoracic wall. This approach enables anatomical reduction and stabilization of the fractured rib fragments, thereby facilitating earlier patient mobilization.^3)^ Several studies in the literature have demonstrated that an appropriately applied SSRF approach can result in favorable clinical outcomes in patients with rib fractures when careful risk assessment is performed. Furthermore, SSRF is associated with improved clinical outcomes, particularly in appropriately selected patients.^5)^

However, posterior rib fractures pose particular challenges, including limited surgical access to the posterior fracture site due to its location beneath the back muscles and the scapula, the complex anatomy of the posterior thoracic wall, the risk of injury to adjacent blood vessels, and the potential for intrathoracic complications. Therefore, their management requires a systematic and evidence-based approach.^3)^ In addition, comprehensive postoperative management, such as adequate pain control, respiratory physiotherapy, and early mobilization, also plays an important role in improving patient prognosis.^5)^

This literature review was conducted to provide a comprehensive understanding of the principles, effectiveness, and limitations of SSRF in posterior rib fractures, as well as the role of postoperative rehabilitation. It is expected that this review will serve as a reference to support improved clinical decision-making and ultimately contribute to better patient outcomes.

## Anatomy and Biomechanical Characteristics of the Posterior Ribs

From an anatomical and biomechanical perspective, the posterior ribs have distinct anatomical and biomechanical characteristics that differentiate them from anterior and lateral segments.^6,7)^ They are closely associated with the vertebral column through the costovertebral and costotransverse joints and are surrounded by thick paraspinal musculature.^6)^ This configuration provides structural stability but also limits surgical accessibility. Compared with anterior ribs, posterior ribs demonstrate reduced respiratory motion but are associated with greater pain due to involvement of the paraspinal muscles and pleura.^6,7)^ In addition, their proximity to the intercostal vessels increases the risk of bleeding and hemothorax in cases of fracture.^8)^

The posterior segment of the ribs has a thicker and stronger structure compared with the anterior portion. This characteristic is related to the articulation of the posterior ribs with the vertebral column and the costovertebral complex. Fractures in this region generally occur as a result of significant rotational or compressive forces. The posterior rib segment also exhibits less respiratory movement compared with the anterior segment, resulting in more limited displacement of fracture fragments. However, fractures of the posterior ribs are often associated with more intense pain due to the involvement of the paraspinal muscles and pleura.^6,7)^

Posterior rib fractures are more frequently associated with a higher risk of bleeding. This is primarily due to their close proximity to the intercostal vessels, which originate from the aorta and course along the inferior border of each rib, gradually decreasing in diameter along their pathway. As a result, fractures in the posterior rib segments may increase the likelihood of vascular injury and bleeding. Consequently, posterior rib fractures can serve as an indicator of more severe thoracic trauma and may influence the overall management strategy.^8)^

## Classification and Characteristics of Posterior Rib Fractures

Posterior rib fractures exhibit distinct biomechanical and clinical characteristics compared with fractures in the anterior and lateral segments. These differences have important implications for injury severity and clinical management, particularly in determining the need for surgical stabilization.^3)^

Based on anatomical location, fractures of the upper ribs^1–3)^ are typically associated with high-energy trauma and major vascular injury.^6,9)^ Middle ribs^4–9)^ are the most commonly affected and are frequently associated with pulmonary complications such as pneumothorax and pulmonary contusion.^7,10)^ Lower rib fractures^10–12)^ may indicate intra-abdominal injury due to their proximity to organs such as the liver, spleen, and kidneys.^9,10)^

## Indications and Contraindications of SSRF

### 1. Indications

The following indications are primarily derived from general rib fracture populations, as posterior-specific evidence remains limited. According to guidelines from the World Society of Emergency Surgery and the Chest Wall Injury Society, the primary indication for SSRF is the presence of FC. Patients with FC frequently present with pulmonary contusions and impaired respiratory mechanics, which can lead to ineffective ventilation and potentially result in respiratory failure. Additional indications include severely displaced rib fractures, with or without the presence of FC.^11,12)^

Other indications include non-FC patients who present with multiple ipsilateral rib fractures (≥3) with severe displacement, as well as multiple rib fractures (≥3) involving ribs 3–10 with displacement, accompanied by respiratory failure despite mechanical ventilation. SSRF may also be considered in patients who are not receiving ventilatory support but exhibit at least 2 pulmonary impairments despite adequate local anesthesia and multimodal analgesia. These impairments include a respiratory rate >20 breaths per minute, reduced oxygen saturation (<90%), or a numeric pain score >5/10.^3,11,13)^

SSRF may also be indicated in patients with rib fractures caused by penetrating injuries, uncontrolled pain despite failure of NOM, and chest wall deformity, particularly when associated with reduced thoracic volume. Additional indications include acute respiratory distress syndrome (ARDS) in patients with chest wall instability, displaced rib fractures identified during thoracotomy performed for other indications, and chronic pain related to rib fracture nonunion.^14)^ Elderly patients with rib fractures have a significantly increased risk of morbidity and mortality following chest wall injury. Studies have demonstrated that, in patients aged >65 years, SSRF can reduce mortality and respiratory complications while also improving respiratory mechanics and facilitating faster functional recovery.^12)^

Posterior rib fractures that impair ventilatory function may also be considered for SSRF. However, this consideration is primarily based on extrapolation from general rib fracture populations and supported by anatomical and biomechanical considerations, rather than direct clinical evidence. Posterior rib fractures located near the costotransverse joint can cause disruption of respiratory mechanics even with minimal instability, thereby increasing the risk of complications such as pneumonia and hemothorax. Therefore, in cases of displaced posterior rib fractures, SSRF is often considered more beneficial than conservative management.^8,12)^

### 2. Contraindications

SSRF is generally contraindicated in patients with hemodynamic instability. In addition, the safety of SSRF in patients with severe pulmonary contusion requiring advanced mechanical ventilation remains uncertain. Some experts suggest that early SSRF may still be considered in selected patients with pulmonary contusion, whereas others regard severe pulmonary contusion as a contraindication to surgical intervention. Furthermore, patients undergoing active resuscitation should not undergo SSRF.^3,12)^

Traumatic brain injury (TBI) is not considered an absolute contraindication to SSRF. Patients with moderate (Glasgow Coma Scale [GCS] 9–12) to severe TBI (GCS ≤8) who have a reasonable prospect of neurological recovery may benefit from the protective effects of SSRF, particularly by reducing the risk of pneumonia and facilitating earlier liberation from mechanical ventilation. However, some guidelines consider TBI, especially severe TBI, to be a contraindication to SSRF. This perspective is based on the concern that the procedure may not provide significant long-term benefits, while the risks associated with anesthesia and surgical intervention may outweigh the potential advantages.^3,11,12)^

In several studies, performing SSRF has been considered high risk in patients with spinal fractures, particularly lower spinal fractures that may result in paraplegia. It is generally recommended that spinal injuries be prioritized and managed first. Performing SSRF prior to spinal surgery in the prone position should only be considered in cases of severe chest wall instability, provided the patient’s neurological status is stable, to reduce the risk of intraoperative complications. Patients with a history of cardiopulmonary comorbidities, uncorrected anticoagulation or coagulopathy, active malignancy, or other terminal illnesses should be carefully evaluated, as these conditions represent relative contraindications to SSRF.^3,14)^

## Principles and Operative Techniques of SSRF

### 1. Basic principles of SSRF in rib fractures

The primary principle of SSRF is to restore the anatomical integrity and functional stability of the chest wall. In posterior rib fractures, this principle is particularly important because the posterior ribs serve as key structural pillars of the thoracic cage. SSRF aims to restore the mechanical stability of the chest wall by reestablishing the segmental rigidity of the thorax, thereby improving ventilatory mechanics. Posterior rib fractures frequently involve angular deformities rather than simple translational displacement; therefore, SSRF plays an important role in achieving adequate anatomical reduction and stabilization of the thoracic wall.^11,15)^

SSRF is performed based on stabilization principles aimed at reducing pain, protecting neurovascular structures, and minimizing trauma to the surrounding soft tissues of the ribs. Surgical stabilization eliminates abnormal motion between fracture fragments, thereby reducing mechanical pain and improving ventilation. It also helps protect the neurovascular structures located along the inferior border of the ribs. In posterior rib fractures, the intercostal nerve lies close to the site of screw placement; therefore, careful screw positioning is required to avoid injury to the intercostal nerve. In addition, trauma to the surrounding soft tissues should be minimized through the use of muscle-sparing surgical techniques.^11,12)^

The timing of SSRF is a crucial consideration in its implementation. Early SSRF offers several advantages, including reducing unfavorable outcomes such as prolonged mechanical ventilation and pneumonia, as well as decreasing the technical complexity of the surgical procedure. Early intervention may also help prevent or limit factors such as severe inflammation, hematoma formation, retained hemothorax, empyema, chest wall stiffness with deformity, and early callus formation. Although many studies support performing SSRF within 72 h after the initial injury, the optimal timing for early SSRF remains a subject of ongoing debate.^3)^

### 2. Operative techniques of SSRF

The operative technique of SSRF consists of several stages. The first step in the procedure is preoperative imaging. The initial evaluation of trauma patients may include a chest radiograph and thoracic ultrasound. However, computed tomography (CT) of the chest, with 2- or 3-dimensional (3D) reconstruction, is often recommended to provide a more accurate diagnosis and better characterization of rib fractures following trauma.^3,10,11)^

CT scan can assist in preoperative planning by enabling measurement of the distance from fixed anatomical landmarks such as the sternum for anterior fractures and the spinous processes for posterior fractures to the site of the rib fracture. In addition, the curvature of the ribs can be accurately assessed and easily identified on CT images. Postoperative CT scans have also been used to demonstrate increased lung volume following SSRF compared with preoperative CT imaging.^10,12,16)^

Ultrasonography may assist in the intraoperative localization of rib fractures, thereby facilitating smaller surgical incisions and potentially reducing operative time. However, these preliminary findings require further validation through additional studies, as there is currently limited evidence comparing the diagnostic accuracy of chest radiography or ultrasonography with CT scanning.^16,13)^ In a study involving patients who underwent SSRF, 3D printing technology was used in approximately one-third of cases to create patient-specific plates prior to surgery and to more accurately estimate the required incision length.^3,10,11)^

Preoperative preparation should be carefully considered. This includes optimizing the patient’s condition by controlling pain, maintaining pulmonary hygiene, evaluating pulmonary function, administering prophylactic antibiotics such as first-generation cephalosporins or equivalent agents, identifying rib fractures, and planning the most appropriate surgical approach and technique. A comprehensive understanding of rib fracture characteristics—such as the degree of displacement, angulation, bone loss, and the number and location of the affected ribs is essential for determining prognosis in patients with thoracic trauma and for assessing the risks and benefits of SSRF.^11,12,16)^

Based on current evidence, stabilization of all displaced ribs through the primary surgical incision is strongly recommended when feasible, as it provides optimal stability, reduces pain, promotes fracture healing, and helps prevent secondary displacement, deformity, and recurrent pain if the fracture progresses to nonunion.^3,5)^ The location of the fracture has important clinical implications because it is closely related to surrounding anatomical structures, such as the presence of costal cartilage in the anterior region and the transverse processes in the posterior region. Fractures involving ribs 1 or 2 are particularly difficult to expose surgically.^16)^ These ribs contribute minimally to respiratory physiology; therefore, fractures involving these ribs are generally not considered candidates for SSRF. Ribs 11 and 12 (floating ribs) also contribute minimally to respiratory mechanics. Posterior rib fractures located within approximately 2.5 cm of the vertebral transverse process are traditionally not considered suitable candidates for fixation. Plate fixation is typically performed only when at least 2 plate holes can be positioned along the rib neck, which requires approximately 20–25 mm of space between the rib head and the tubercle. Therefore, ribs 3 to 10 are the most common targets for surgical stabilization.^3,11,12)^

Posterior and lateral rib fractures can be exposed through the auscultatory triangle, which is bordered by the medial border of the scapula, the latissimus dorsi, and the trapezius muscle. Exposure is typically achieved using a muscle-sparing technique by separating the latissimus dorsi from the trapezius rather than transecting these muscles. The patient is usually positioned in the lateral decubitus position. In cases of bilateral rib fractures, the ipsilateral side is generally addressed first, after which the patient may be repositioned to treat the contralateral side, either during the same operation or in a staged procedure. The specific incision is determined according to the fracture pattern identified on preoperative imaging. Common approaches include a vertical axillary incision or a lateral thoracic incision. In some cases, the patient may be positioned in the prone position to allow better access and visualization of posterior rib fractures. Additional techniques that may facilitate access to difficult-to-reach fractures include percutaneous trocar access, the use of a hanging scapula retractor, stepladder incisions, and the use of low-profile 90° screwdrivers and drills.^5,12)^

Anterior rib fractures can be accessed through an inframammary incision. The patient is positioned in the supine position, with the arms supported using a candy cane stirrup or placed on arm boards. This positioning allows bilateral anterior rib fractures to be repaired without the need for patient repositioning. This approach should employ a muscle-sparing technique by elevating the inferior portion of the pectoralis muscle, followed by blunt dissection in the subpectoral plane while carefully avoiding injury to the surrounding nerves.^5)^

Reduction and countertraction can be performed using various clamps available in specialized rib fracture instrument sets; however, a towel clamp is often the most effective instrument because it provides gentle elevating pressure on the fracture segment. Fixation plates should be applied flush to the surface of the rib. Although most plating systems are pre-contoured, additional modification is often required to ensure an optimal fit. Fixation may be performed using either unicortical or bicortical techniques, depending on the system used. In bicortical fixation, accurate measurement of rib thickness is necessary to ensure appropriate screw length selection. In addition, plates should ideally be positioned along the superior two-thirds of the rib to minimize the risk of injury to the neurovascular bundle. After completion of the SSRF procedure, wound closure can be performed using staples or cosmetic sutures, depending on the location of the incision, the condition of the surrounding tissues, and cosmetic considerations.^5,11)^

## Effectiveness of SSRF

Currently, there is a lack of direct clinical evidence specifically evaluating outcomes of SSRF in posterior rib fractures, including key clinical endpoints such as mortality, intensive care unit (ICU) length of stay, duration of mechanical ventilation, and complication rates. The effectiveness of SSRF has been demonstrated in numerous studies involving rib fractures in general, particularly in patients with FC. However, evidence specifically addressing posterior rib fractures remains limited. Therefore, the clinical benefits of SSRF in posterior rib fractures are largely extrapolated from broader SSRF studies and should be interpreted with caution.

In general rib fracture populations, SSRF has been associated with improved clinical outcomes compared with nonoperative management, including reduced duration of mechanical ventilation, decreased incidence of pneumonia, and shorter ICU and hospital length of stay.^3,16)^ These benefits are particularly evident in patients with FC, where stabilization of the chest wall improves respiratory mechanics and reduces pulmonary complications. Mechanistically, SSRF stabilizes fracture segments, reduces pain during respiration, and improves ventilation, thereby facilitating effective airway clearance and early mobilization. These effects contribute to a reduction in pulmonary complications and overall improved recovery.^11,17)^

A review by the Eastern Association for the Surgery of Trauma, which included 22 studies involving 986 patients, demonstrated that SSRF was associated with lower morbidity, reduced duration of mechanical ventilation, shorter ICU and hospital stay, and decreased incidence of pneumonia and tracheostomy.^18)^ Similarly, timing of intervention appears to influence outcomes. Dixon *et al*. reported that pneumonia occurred in 18% of patients who underwent SSRF within 72 h compared with 58% in those who received delayed stabilization.^19)^

In elderly patients, SSRF has also been associated with improved outcomes in selected cases. Large cohort studies have demonstrated reductions in mortality and respiratory complications, although these benefits may be accompanied by increased complication rates and longer hospital stay in some populations.^20,21)^

Despite these findings, it is important to emphasize that most of the available evidence is derived from studies involving general rib fractures or FC rather than posterior rib fractures specifically. Given the distinct anatomical and technical challenges associated with posterior rib fractures, the direct applicability of these findings remains uncertain, highlighting the need for posterior-specific studies.

## Limitations and Challenges of SSRF in Posterior Rib Fractures

Despite the established benefits of SSRF in the management of rib fractures, several limitations remain, particularly in the context of posterior rib fractures.^3,11)^ One of the most important limitations is the lack of high-quality evidence specifically addressing posterior rib fractures. Most of the available data are derived from studies involving general rib fractures or FC, which may not fully reflect the unique anatomical and technical considerations of posterior injuries.^3,22)^

From an anatomical perspective, these fractures present specific challenges due to their proximity to the thoracic vertebrae, scapula, and thick paraspinal musculature.^6,7)^ These factors limit surgical exposure and increase procedural complexity, often requiring specialized approaches such as muscle-sparing techniques or alternative patient positioning.^5,12)^ In addition, the posterior rib segments are closely related to the intercostal neurovascular bundle, increasing the risk of iatrogenic injury during fixation.^11,12)^ Accurate screw placement and careful operative planning are therefore essential to minimize complications such as chronic pain or sensory disturbances.

Another important limitation is the variability in clinical practice and resource availability. SSRF is typically performed in specialized centers with appropriate surgical expertise and instrumentation, which may limit its widespread implementation.^3)^ Furthermore, differences in patient characteristics, injury patterns, and institutional protocols contribute to heterogeneity in reported outcomes.

Taken together, these limitations highlight that current evidence supporting SSRF cannot be directly generalized to posterior rib fractures. Therefore, careful patient selection and individualized clinical decision-making remain essential in this subgroup.

## Postoperative Rehabilitation after SSRF

Postoperative rehabilitation after SSRF is an important component in supporting the success of SSRF, particularly with respect to recovery of respiratory function and patient mobilization.^3)^ Rehabilitation is generally multidisciplinary and involves thoracic surgeons, rehabilitation physicians, physiotherapists, and intensive care teams. The primary goals of rehabilitation are to restore ventilatory function, reduce pain, prevent pulmonary complications, and accelerate patient mobilization.^23,24)^

The main intervention for pulmonary recovery after SSRF is pulmonary rehabilitation. These programs include diaphragmatic breathing exercises, incentive spirometry, and effective coughing exercises. Diaphragmatic breathing helps improve alveolar ventilation, incentive spirometry encourages deep inspiration and increases lung expansion, and effective coughing exercises help clear secretions from the airways. Routine implementation of pulmonary rehabilitation has been shown to reduce the incidence of atelectasis and pneumonia and to accelerate functional recovery in thoracic trauma patients.^22,23,25)^

In patients after SSRF, multimodal analgesia should be considered a fundamental aspect of postoperative rehabilitation for pain control. Pain resulting from thoracic trauma may inhibit lung expansion, reduce the ability to cough effectively, and increase the risk of complications such as atelectasis and pneumonia.^23)^ Pain management includes combinations of systemic analgesics (opioids and non-opioids), nonsteroidal anti-inflammatory drugs, paracetamol, and regional techniques such as epidural analgesia or intercostal nerve blocks. Transcutaneous electrical nerve stimulation (TENS), using low frequencies (<10 Hz) to high frequencies (>50 Hz), may activate sensory nerves that stimulate the gate-control mechanism and the opioid system, thereby reducing pain. Low-frequency TENS activates μ-opioid receptors, whereas high-frequency TENS activates δ-opioid receptors in the spinal cord and brainstem. The combination of analgesic medications and TENS is more effective in reducing pain.^26)^ When pain control is implemented optimally and adequately, patients are better able to breathe deeply, actively participate in respiratory physiotherapy, and improve early mobilization.^3,5,26)^

Early mobilization after cardiothoracic surgery has been shown to reduce the duration of hospitalization.^24)^ After SSRF, patients are encouraged to begin activity gradually, starting from sitting on the bed, then standing, and eventually walking. The benefits of early mobilization include improved pulmonary ventilation and perfusion, maintenance of muscle function, and preservation of cardiovascular function. Additional benefits include prevention of deep vein thrombosis and thromboembolic complications.^23,22)^

Rehabilitation after SSRF varies among patients depending on several factors, including the number of fractures, severity of trauma, pulmonary condition, and the presence of associated injuries.^12)^ In the early phase, the main focus is pain control, breathing exercises, and light mobilization.^23,27)^ This is followed by the next phase, during which the patient begins more active functional exercises, including progressive physical and muscle training. The final phase is advanced rehabilitation, which focuses on restoring physical capacity and respiratory function optimally until the patient is considered ready to return to normal activities.^23,22,27)^

## Technological Advances and Current Research Gaps in SSRF

Technological advancements in SSRF over the past few decades have progressed rapidly, particularly in operative techniques, implant design, and imaging-based preoperative planning methods.^22)^ These advancements aim to improve the accuracy of fracture reduction, facilitate surgical access, particularly for posterior rib fractures that are anatomically difficult to reach, and reduce operative morbidity. Several studies have demonstrated that the application of minimally invasive techniques can reduce soft tissue dissection, which is associated with faster patient recovery and decreased postoperative pain. These benefits are especially significant for elderly patients and those with comorbidities. However, despite the rapid advancement of technology, further research is still needed to support the development and refinement of clinical practice in the future.^5,12,28)^

A notable technological innovation in SSRF is the development of minimally invasive rib fixation techniques. This technique utilizes smaller incisions and muscle-sparing approaches to reduce damage to the surrounding soft tissues. It is often combined with thoracoscopic or endoscopic assistance to improve visualization of rib fractures within the thoracic cavity.^11,29)^ Video-assisted thoracoscopic surgery (VATS) is increasingly utilized in the management of rib fractures. With the use of VATS, rib fractures can be reduced and plated on the inner cortex of the rib under direct visualization. VATS also allows optimal placement of surgical incisions to minimize incision length and muscle division. In addition, it facilitates complete evacuation of hemothorax, optimizes chest tube placement, assists in fracture reduction, and reduces the risk of injury to the lungs and diaphragm.^5,11,12,29)^

Patients with severe thoracic trauma generally have higher Injury Severity Scores (ISS), and the safety and effectiveness of SSRF performed using VATS in such patients, including those with severe thoracic trauma such as FC, remain uncertain. However, most patients who underwent SSRF via VATS in previously reported studies had an ISS of less than 16. Despite the relatively low ISS in these cases, SSRF using VATS still has certain limitations, particularly in patients with bilateral pulmonary contusions, which are commonly observed in patients with severe bilateral rib fractures. In such cases, prolonged single-lung ventilation during SSRF with VATS may be required, which could increase the risk of postoperative pulmonary complications.^29)^

To further reduce surgical trauma associated with chest wall stabilization, minimally invasive plate osteosynthesis (MIPO) has been introduced. This technique involves the use of smaller, fracture-focused incisions to minimize soft tissue injury and better preserve the local blood supply.^28)^ MIPO may be considered in patients with a single rib fracture, particularly within the context of research studies.^3,11)^ In the MIPO procedure, after separation of the subcutaneous tissue and muscle fibers, a cavity is created between the chest wall and the overlying soft tissue, allowing the placement of a retractor. An inner ring is then positioned between the chest wall and the soft tissue, while the retractor is secured to an outer ring, generating outward traction and creating a working window through which the procedure can be performed. In addition, a rubber seal located between the inner and outer rings provides a hemostatic effect, helping to maintain a dry operative field.^3)^

The minimally invasive tunnel technique is another promising alternative as technological advancements continue to evolve in the field of SSRF. This approach involves small incisions and the use of specialized instruments, aiming to achieve outcomes comparable to or superior to those of traditional methods while minimizing surgical trauma. By creating a narrow tunnel above the fracture site, the surgeon can insert screws and plates without extensive exposure, thereby reducing injury to the surrounding muscles, nerves, and blood vessels.^28)^ Recent biomechanical studies have also focused on evaluating the mechanical strength of fixation systems used in rib fractures, particularly posterior rib fractures. A study conducted by Pieracci et al. demonstrated that the use of a locking plate system provides better stabilization compared with conventional fixation methods.^22)^ In addition, biomechanical research has evaluated various screw configurations and plate positions to determine the most optimal technique for withstanding the continuous respiratory forces acting on the chest wall. These findings contribute to the development of stronger implant designs that are better suited to the biomechanical demands of the posterior thorax.^14)^

Advances in medical technology, including the use of artificial intelligence (AI), have shown potential to improve the evaluation and management of thoracic trauma. The ability of AI to rapidly analyze imaging data in real time allows for the early identification of rib fractures, supports triage processes, and helps determine the priority of interventions, including indications for SSRF. Furthermore, the integration of imaging data, vital signs, and trauma scores enables stratification of injury severity and prediction of complications such as ARDS, thereby potentially improving the accuracy of clinical decision-making and surgical planning.^13)^

Despite ongoing technological advancements in SSRF, research gaps remain, particularly in relation to posterior rib fractures. Most of the scientific evidence supporting SSRF is derived from studies involving FC or anterior and lateral rib fractures, whereas studies specifically evaluating posterior rib fractures are still limited.^22)^ Given these gaps in the current evidence, further studies are required to strengthen the scientific evidence regarding the application of SSRF in posterior rib fractures. Prospective multicenter studies with robust methodological designs are expected to provide clearer recommendations on the appropriate indications, optimal surgical techniques, and long-term outcomes in this patient population.^18,22,29)^

## Discussion and Future Directions

This review highlights that, although SSRF has been widely recognized as an effective intervention for rib fractures in general, its role in posterior rib fractures remains less clearly defined.^3,18)^ The majority of current evidence is derived from studies involving anterior and lateral rib fractures or FC, whereas posterior-specific data remain limited.^18,22)^

Given the unique anatomical and biomechanical characteristics of posterior rib fractures, it is likely that their optimal management differs from that of other rib fracture patterns.^6,7)^ Factors such as limited surgical access, proximity to the vertebral column, and increased technical complexity must be carefully considered when evaluating the role of SSRF in this subgroup.^5,12)^

Future research should focus on prospective and multicenter studies specifically evaluating posterior rib fractures. These studies are needed to define clearer indications, optimize surgical techniques, and evaluate long-term outcomes.^22)^ Advances in imaging, minimally invasive techniques, and patient-specific surgical planning may further improve the safety and feasibility of SSRF in this population.^3,29)^

Addressing these gaps is essential to strengthen the evidence base and to guide more precise and individualized clinical decision-making in the management of posterior rib fractures. Therefore, current recommendations for SSRF in posterior rib fractures should be interpreted as extrapolated evidence combined with anatomical and biomechanical considerations, rather than as direct clinical validation.

## Conclusions

Posterior rib fractures represent a form of thoracic injury that can lead to serious complications due to their proximity to vital structures, including the intercostal vessels, lungs, and other intrathoracic organs. This condition may disrupt respiratory mechanics and increase the risk of complications such as pneumothorax, hemothorax, and atelectasis, particularly in cases of multiple rib fractures or when associated with FC.

SSRF has been shown to provide clinical benefits in patients with appropriate indications, including reducing the duration of mechanical ventilation, decreasing pulmonary complications, and facilitating earlier patient mobilization. However, the application of SSRF in posterior rib fractures presents unique challenges related to anatomical complexity, which may make surgical access more difficult. Therefore, careful patient selection and adequate surgical expertise are essential to achieve optimal outcomes.

Treatment outcomes depend not only on the surgical procedure but also on comprehensive postoperative rehabilitation, including adequate pain control, respiratory physiotherapy, and early mobilization to optimize respiratory recovery. Although surgical technologies and techniques continue to advance, scientific evidence specifically addressing SSRF in posterior rib fractures remains limited. Therefore, further research is required to strengthen the scientific evidence and inform clinical guidelines for its management.

## Declarations

### Data availability

Data sharing is not applicable to this article as no datasets were generated or analyzed during the current study.
